# Synergy between CD8 T Cells and Th1 or Th2 Polarised CD4 T Cells for Adoptive Immunotherapy of Brain Tumours

**DOI:** 10.1371/journal.pone.0063933

**Published:** 2013-05-23

**Authors:** Sabine Hoepner, Jacelyn M. S. Loh, Cristina Riccadonna, Madiha Derouazi, Céline Yacoub Maroun, Pierre-Yves Dietrich, Paul R. Walker

**Affiliations:** Centre of Oncology, Geneva University Hospitals and University of Geneva, Geneva, Switzerland; The University of Chicago, United States of America

## Abstract

The feasibility of cancer immunotherapy mediated by T lymphocytes is now a clinical reality. Indeed, many tumour associated antigens have been identified for cytotoxic CD8 T cells, which are believed to be key mediators of tumour rejection. However, for aggressive malignancies in specialised anatomic sites such as the brain, a limiting factor is suboptimal tumour infiltration by CD8 T cells. Here we take advantage of recent advances in T cell biology to differentially polarise CD4 T cells in order to explore their capacity to enhance immunotherapy. We used an adoptive cell therapy approach to work with clonal T cell populations of defined specificity. Th1 CD4 T cells preferentially homed to and accumulated within intracranial tumours compared with Th2 CD4 T cells. Moreover, tumour-antigen specific Th1 CD4 T cells enhanced CD8 T cell recruitment and function within the brain tumour bed. Survival of mice bearing intracranial tumours was significantly prolonged when CD4 and CD8 T cells were co-transferred. These results should encourage further definition of tumour antigens recognised by CD4 T cells, and exploitation of both CD4 and CD8 T cell subsets to optimise T cell therapy of cancer.

## Introduction

After decades of advances in fundamental and applied tumour immunology, the potential of the immune system to treat patients with cancer has now been validated in several landmark clinical trials [1]. However, how to optimally exploit effector T cells to eradicate tumour cells remains a major challenge because of the complexity of orchestrating immune interactions in lymphoid organs as well as at the tumour site of the patient. An efficacious cancer vaccine must achieve this, but there are alternative strategies. One appealing approach in development is to use adoptive T cell therapy, in which tumour-specific T cells can be optimally stimulated and expanded in vitro and then reinfused into the patient to hopefully destroy the tumour [2]. Most of these studies have involved transfer of CD8 T cells that can differentiate into potent cytotoxic T lymphocytes (CTLs) and directly recognise antigens presented on Major Histocompatibility Complex (MHC) class I molecules expressed by tumour cells. Some clinical trials for advanced malignancies have confirmed the potential of this approach to achieve prolonged remission in certain patients, although this required heavy lymphodepletion before T cell transfer [3], or engineering of CD8 T cells to improve tumour recognition [4].

Brain malignancies present special challenges for conventional treatment modalities because of their localisation in a specialised anatomic site in which surgical resection is not always feasible, or never complete for infiltrative tumours like glioblastoma. Moreover, even for relatively chemo- and radiosensitive central nervous system (CNS) lymphomas, these treatments are not ideal because of the risk of neurotoxic effects [5]. However, T-cell mediated immunotherapy is a highly attractive approach because of the capacity of T cells to infiltrate the brain and to specifically destroy cancer cells with little collateral damage to critical neural tissue, as confirmed in many preclinical studies [6]. In patients with malignant brain tumours, the degree of intratumoural effector T cell infiltration has been correlated with longer survival of patients with glioblastoma [7], and many early phase immunotherapy trials show promising results for some patients [8], [9]. Ultimately, the success of T cell-mediated immunotherapy will depend upon sufficient effector T cells infiltrating the brain tumour [7]. Here they must override regulatory cells and molecules, particularly regulatory T cells (Tregs) and transforming growth factor (TGF)-β; these are essential for immune homeostasis of healthy tissue, but they severely attenuate anti-tumour immunity [10], [11]. Importantly for immunotherapy design, depletion or neutralisation of Tregs or TGF-β was not an absolute requirement to elicit T cell effector functions, since strong immune stimuli alone could restore immune function in murine brain tumour models [12], [13].

Efficacious T cell immunotherapy for brain tumours requires an understanding of how T cells can home to the tumour site. Physiologically, when T cells are activated in vivo by antigen presenting cells (APCs), they are also imprinted with homing receptors (adhesion molecules and chemokine receptors) that facilitate preferential entry to different tissues [14]. For T cell migration to brain tumours, very-late antigen (VLA)-4 (α4β1 integrin) and CXCR3 are particularly well defined as playing key roles for tumour-reactive CD8 T cells [15], [16], [17]. For CD4 T cells, their migration to the brain has been mostly studied in the context of encephalitogenic T cells. Indeed, as for CD8 T cells, α4β1 integrin is implicated for CD4 T cell migration, and is a therapeutic target in multiple sclerosis [18].

Despite the anti-tumour potential of CD4 T cells, they are currently underexploited because of uncertainties about generating cells in vitro with the capacity to home to the tumour site and exert appropriate in vivo functions. One complexity is the plasticity of CD4 T cells: after activation in a particular cytokine milieu they can be polarised towards multiple helper lineages (including Th1, Th2, Th17) based on cytokine secretion profiles, or towards induced regulatory T cells (iTreg) with suppressive function [19]. CD4 polarisation status has not always been defined in tumour immunology studies, but both Th1 and Th2 polarised T cells have been reported to have anti-tumour function [20], [21]. More recently, Th17 cells were also proposed to have strong anti-tumour activity [22], although their use for brain tumour therapy could be risky in view of the strong association of this subset with autoimmune neuroinflammation [23]. Cytokine polarised CD4 Th cells not only have a different cytokine secretion profile, but they also express different chemokine receptors and integrins, which mediate tissue selective migration [24]. Expression of CCR5 and CXCR3 is particularly associated with a Th1 phenotype, while CCR4 and CCR8 are associated with a Th2 phenotype [25], [26]. For integrins, Th1 CD4 T cells showed a higher expression of VLA-4 and VLA-6 (α6β1 integrin) than Th2 cells, and the Th1 cells displayed preferential tumour homing and therapeutic effect in a subcutaneous melanoma model [27].

In this study we investigated polarisation of tumour-reactive CD4 T cells and its impact on homing to the brain of mice bearing an intracranial tumour. The potential of combined CD4 and CD8 T cell transfer in brain tumour immunotherapy was previously highlighted in an intracranial fibrosarcoma model, although polarisation status was not studied [28]. Here, we used in vitro polarisation of CD4 T cells towards Th1 or Th2 lineages, which resulted in different patterns of homing receptor expression. Th1 cells expressed high levels of α4 integrin and CXCR3 and homed more efficiently to the brain of tumour bearing mice than Th2 cells. Moreover, when Th1 cells were tumour-specific, they promoted recruitment of CD8 T cells to the brain, and enhanced their function. Finally, in adoptive transfer therapy of an intracranial tumour, both Th1 and Th2 polarised T cells significantly enhanced survival when co-transferred with CD8 T cells.

## Materials and Methods

### Mice

Female C57BL/6J mice (CD45.2) were purchased from Charles River Laboratories (L'Arbresle, France). T cell receptor (TCR) transgenic mice were all on a C57BL/6 background, but in some cases expressed congenic markers used for their identification after adoptive transfer (CD45.1 or Thy1.1). P14 transgenic mice which express a Vα2/Vβ8.1 TCR directed against MHC class I restricted epitope lymphocytic choriomeningitis virus (LCMV)-GP_33–41_ were kindly provided by H. Pircher (Freiburg, Germany). SMARTA TCR transgenic mice, expressing a TCR for the MHC II restricted epitope LCMV-GP_61–80_ were kindly provided by P. Ohashi (Toronto, Canada). OTI and OTII mice express TCR specific for Ovalbumin (OVA) epitopes restricted by MHC class I and MHC class II, respectively (OTI: OVA_257–264_; OTII: OVA_323–339_). They were kindly provided by P. Romero (Lausanne, Switzerland) and T. Schüler (Berlin, Germany). All animals used in this study were between 6 and 10 weeks of age at the time of experiments. These studies have been reviewed and approved by the institutional and cantonal veterinary authorities (Direction Générale de la Santé, République et Canton de Genève, authorisation: 1064/3717/2) in accordance with Swiss Federal law on animal protection.

### Cell Isolation

For isolation of brain infiltrating leucocytes (BILs), tumour implanted and adoptively transferred mice were transcardially perfused with isotonic Ringer’s solution, brains were removed and BILs were isolated as previous described [13]. Immune cells from TCR transgenic mice were obtained from pooled spleens and lymph nodes; they were not further purified prior to in vitro stimulation.

### Cell Lines

The MC57-GP fibrosarcoma (C57BL/6 origin, [29]) was kindly provided by R.M. Zinkernagel (Zürich, Switzerland); it stably expresses the complete LCMV glycoprotein (LCMV-GP). The EG-7 lymphoma (C57BL/6 origin, CRL-2113™, American Type Culture Collection, Manassas, VA) stably expresses full length OVA.

### T cell Polarising Cultures

Two different protocols were used to polarise CD4 T cells, for both, the basic culture medium was DMEM/6% foetal calf serum/20 µM 2-ME/100 U/ml Interleukin (IL)-2. 1 µM GP_61–80_ peptide was added for SMARTA T cells, and 1 µM OVA_323–339_ peptide was added for OTII T cells. For Th1 polarisation, we added 1 µg/ml anti-IL-4 (Biolegend, San Diego, CA) and 2 ng/ml IL-12 (Immunotools, Friesoythe, Germany). For Th2 polarisation, we added 5 µg/ml anti-IFNγ (Biolegend) and 100 ng/ml IL-4. CD4 T cells proliferated to account for >98% of the culture by day 7 when they were used for adoptive transfer experiments.

### Adoptive Transfer of Transgenic T cells

Seven days after in vitro activation, CD62L^+^ cells were eliminated from the activated cell mix using anti-CD62L-PE antibody (Biolegend) and anti-PE magnetic bead separation (Miltenyi Biotech, Bergisch Gladbach, Germany). Fluorescent labelling of cells was performed using either CellTrace Violet proliferation kit (Invitrogen-Life Technologies, Carlsbad, CA), as described in the manufacturer’s protocol, or with 10 µM 5-(and 6) Carboxyfluorescein diacetate succinimidyl ester (CFSE) (Invitrogen) for 5 min at room temperature. Preliminary experiments indicated that choice of dye did not influence function or phenotype of the T cells. For tumour survival experiments, OTII lymphocytes were used without fluorescent labeling. For the adoptive transfer, mice were infused intravenously in phosphate buffered saline. In experiments combining CD4 and CD8 T cells, CD8 T cells were either used activated for 5 days with 1 µM peptide and 100 U/ml IL-2 and infused simultaneously for survival experiments, or were administered naïve and one day prior to CD4 T cells for brain accumulation experiments.

### Tumour Cell Implantations and Survival

Implantation in the brain of recipient mice was performed with a stereotaxic apparatus as previously described [15] using 4×10^5^ MC57-GP cells in 4 µl of methylcellulose. For EG-7, 5×10^5^ cells in 5 ul methylcellulose were injected. Recipient mice were injected with tumour cells 2 days (MC57-GP) or 6 days (EG-7) before adoptive transfer. For homing assays, tumour cells were injected 4 days before intravenous injection of polarised CD4 T cells. Animals used in survival experiments were monitored daily for the manifestation of any pathological signs and weight loss, and were sacrificed according to the criteria authorised by the veterinary authorities (20% weight loss and/or presence of adverse symptoms).

### Antibodies and Flow Cytometry

For intracellular staining, cells were ex vivo restimulated with 5 µg/ml specific peptides and 2 µg/ml anti-CD28 for 5 hours at 37°C, 8% CO2. For restimulation of in vitro polarised cells, restimulation was with phorbol myristate acetate at 100 ng/ml and ionomycin at 1 µg/ml. Brefeldin A (5 µg/ml) was added after the first hour of incubation. After 5 hours cells were harvested and incubated with LIVE/DEAD® Fixable Dead Cell Stain Kit as described in manufacturer’s protocol (Invitrogen). After blocking the Fc receptor binding, the following antibodies for surface staining were used; CD8 (53–6.7), CD4 (GK1.5), CD62L (MEL-14), CD49d (α4-intergrin, PS/2), CD45.1 (A20), CXCR3 (CXCR3-173), CCR4 (2G12), CD45.2 (104), Vβ5.1/5.2 (MR9-4). After surface staining, cells were prepared for intracellular staining by using BD Biosciences (Franklin Lakes, NJ) Cytofix/Cytoperm kit as described in manufacturer’s protocol. The following antibodies were used for intracellular staining; IFN-γ (XMG1.2), IL-4 (11B11), tumour necrosis factor (TNF)-α (TN3-19.12). All antibodies were purchased from either BD Biosciences or Biolegend. Live gated cells were analysed on a Gallios flow cytometer (Beckman Coulter, Brea, CA) and collected data was analysed for antigen expression and cell number (used in estimating absolute cell numbers) using Kaluza software (Beckman Coulter).

### Statistical Analysis

Differences between groups were analysed by either the Mann-Whitney Rank sum test or by the Student’s t test, according to the distribution of the data. Values<0.05 (*), <0.01 (**), <0.001 (***) were considered as significant.

## Results

### Th1 and Th2 Polarisation Leads to Differential Homing Receptor Expression

**Figure 1:**
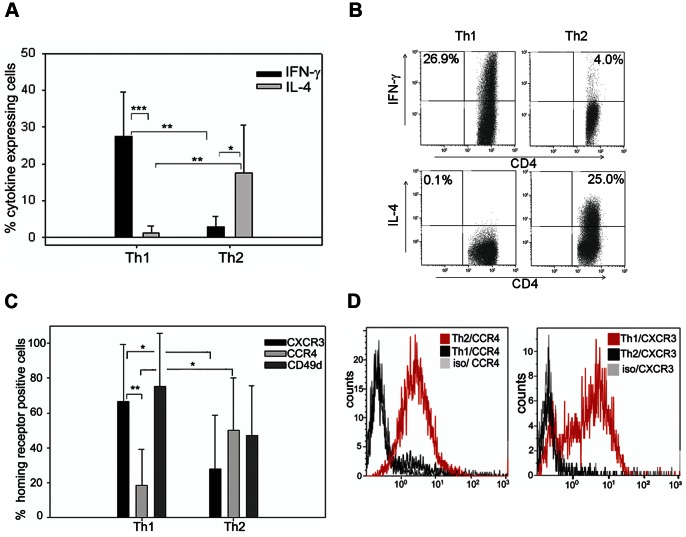
Distinct phenotypes of CD4 T cells activated under Th1 and Th2 polarising conditions. (A) Intracellular staining of polarised T cells for IFN-γ and IL-4. Bars indicate percentage cytokine expressing CD4 gated T cells after flow cytometric analysis, error bars represent SD. *P<0.05, **P<0.01, ***P<0.001, t-test, n = 8. (B) Illustrative intracellular staining for IFN-γ and IL-4 (quadrants set according to isotype control) from Th1 and Th2 polarised T cells. (C) Surface staining and flow cytometry analysis of cell surface receptors implicated in lymphocyte homing; pooled data for expression of chemokine receptors CXCR3, CCR4, and CD49d. Bars indicate percentage homing receptor expressing CD4 gated T cells after flow cytometric analysis, error bars represent SD. *P<0.05, **P<0.01, t-test, n = 8. (D) Representative staining of chemokine receptors CCR4 and CXCR3 on Th2 and Th1 polarised T cells.

We investigated whether in vitro Th1 and Th2 polarisation could generate CD4 T cells with functional properties useful for adoptive therapy of brain tumours. We used clonal populations of T cells from TCR transgenic mice to have T cells bearing identical TCRs that differed only in their polarisation status. CD4 T cells from SMARTA mice are specific for the LCMV epitope GP_61–80_. We isolated spleen and lymph node cells from SMARTA mice and stimulated them in vitro using standard polarising conditions: IL-2, IL-12, and anti-IL-4 for Th1; IL-2, IL-4 and anti-IFN-γ for Th2. We evaluated expression of the key cytokines characterising the Th1 and Th2 dichotomy and confirmed significant IFN-γ with negligible IL-4 expression in Th1 polarising conditions, and IL-4 expression with little IFN-γ in Th2 polarising conditions (Figure 1A, B); although absolute levels of cytokines were variable, the overall skewing towards either IFN-γ or IL-4 expression was highly reproducible. These polarising conditions did not result in IL-17 or Foxp3 expression in either population (data not shown). Hereafter, polarised CD4 T cells are referred to simply as Th1 or Th2 cells according to the polarising protocol they were subjected to. We also applied the same polarising protocols to CD4 T cells from OTII mice, specific for the ovalbumin (OVA) epitope OVA_323–239_, with similar results for IFN-γ and IL-4 expression (Figure S1a). We then examined the expression pattern of homing receptors, since they are influenced by the cytokine milieu. We measured expression of CXCR3, CCR4 as well as that of the α4-integrin subunit (CD49d) of VLA-4. Th1 polarised SMARTA cells were characterised by significantly higher CXCR3 and lower CCR4 expression than Th2 polarised cells; CD49d expression was also higher in Th1 polarised cells, although this did not reach statistical significance (Figure 1C, D). The same association of chemokine receptor and integrin expression patterns with Th1 and Th2 polarisation was also found in OVA-specific OTII CD4 T cells (Figure S1b). Taken together, these results indicate that we are able to generate clonal populations of CD4 T cells from two different models (with two different antigen-specificities), which are predicted to have different effector functions and migratory properties based on cytokine secretion and homing receptor expression.

### Th1 Cells Infiltrate Brain Tumours more Efficiently than Th2 Cells

**Figure 2:**
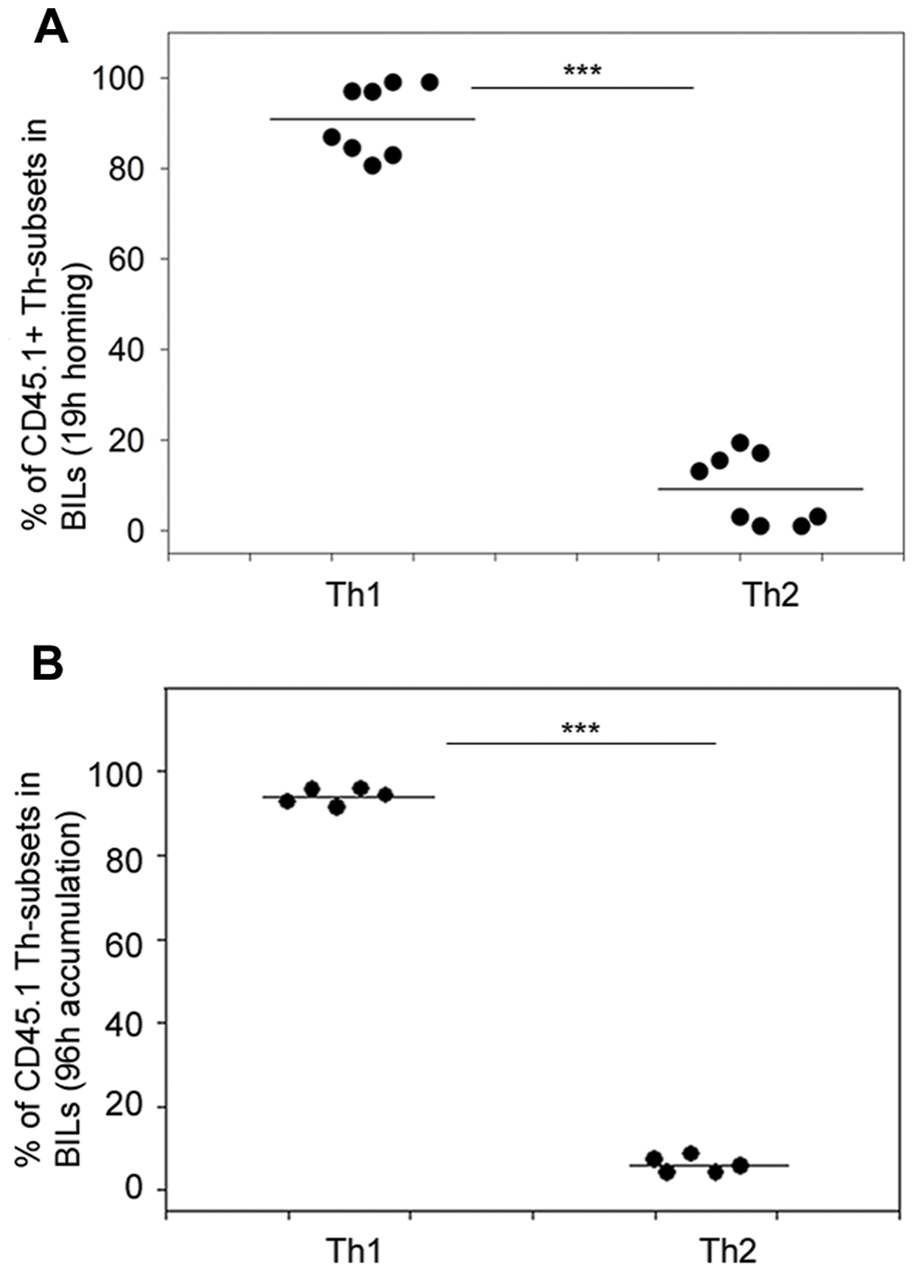
Adoptively transferred Th1 cells show preferential homing and accumulation in an intracranial tumour compared with Th2 cells. SMARTA T cells (CD45.1) were labelled with CFSE (Th1) or Violet dye (Th2) and were intravenously transferred (3×10^6^ Th1; 3×10^6^ Th2) into C57BL/6 mice (CD45.2) that had been intracranially implanted with 4×10^5^ MC57-GP tumour cells 4 days previously. After 19 hours (A), or 96 hours (B), BILs were isolated, stained with antibodies for CD4 and CD45.1 and were analysed ex vivo by multicolour flow cytometry. Adoptively transferred T cells were identified as CD45.1^+^CD4^+^ cells that were either CFSE^+^ or Violet dye^+^ (supporting information Figure S2). Results are expressed as the percentage of Th1 and Th2 cells among the adoptively transferred CD45.1^+^CD4^+^ cells in the BILs, each symbol represents an individual mouse (n = 8). ***P<0.001, *t-*test.

We then investigated the potential of adoptively transferred in vitro polarised tumour-specific Th1 and Th2 cells to home to and accumulate within a brain tumour. We intravenously injected a mixed cell suspension containing equal numbers of dye-labelled Th1 and Th2 SMARTA T cells into syngeneic C57BL/6 mice bearing an intracranial tumour that had been implanted 4 days previously. We used the MC57-GP tumour that expresses the LCMV-glycoprotein recognised by SMARTA T cells. We first assessed homing 19 hours after adoptive transfer by quantifying T cells infiltrating the brain (Figure 2A). Importantly, we thoroughly perfused mice before sacrifice to eliminate leukocytes present in the blood, ensuring that we accurately analysed only BILs. Discrimination of adoptively transferred cells from host T cells, and identification of Th1 and Th2 cells, was precise and unambiguous by use of the CD45.1 congenic marker on transferred cells, and fluorescent dye labelling (Figure S2). The vast majority (approximately 80–100%) of adoptively transferred SMARTA T cells detected at the brain tumour site were Th1 cells (Figure 2a). This finding was not unique to this combination of TCR transgenic mouse and tumour model, since we obtained similar results for Th1 and Th2 cells from OTII mice (Figure S3). For this OTII adoptive transfer, more than 90% of T cells infiltrating the brains of these mice were Th1 cells in the short term homing assay.

T cell migration, entry and exit from tissues is a dynamic process; for the CNS, rapidly infiltrating T cells can leave equally rapidly [30]. We therefore verified whether the preferential Th1 brain tumour infiltration was a transient phenomenon and whether Th2 cells appeared at a later time point (4 days) (Figure 2B). This test therefore measures net accumulation of each cell population over the time period, as well as taking into account T cells that may have exited the brain. The results indicate the same highly significant (P<0.001) accumulation of Th1 cells as found in the short term homing assay.

### Adoptively Transferred Antigen-specific Th1 cells but not Th2 Promote CD8 T cell Recruitment into Brain Tumours

**Figure 3:**
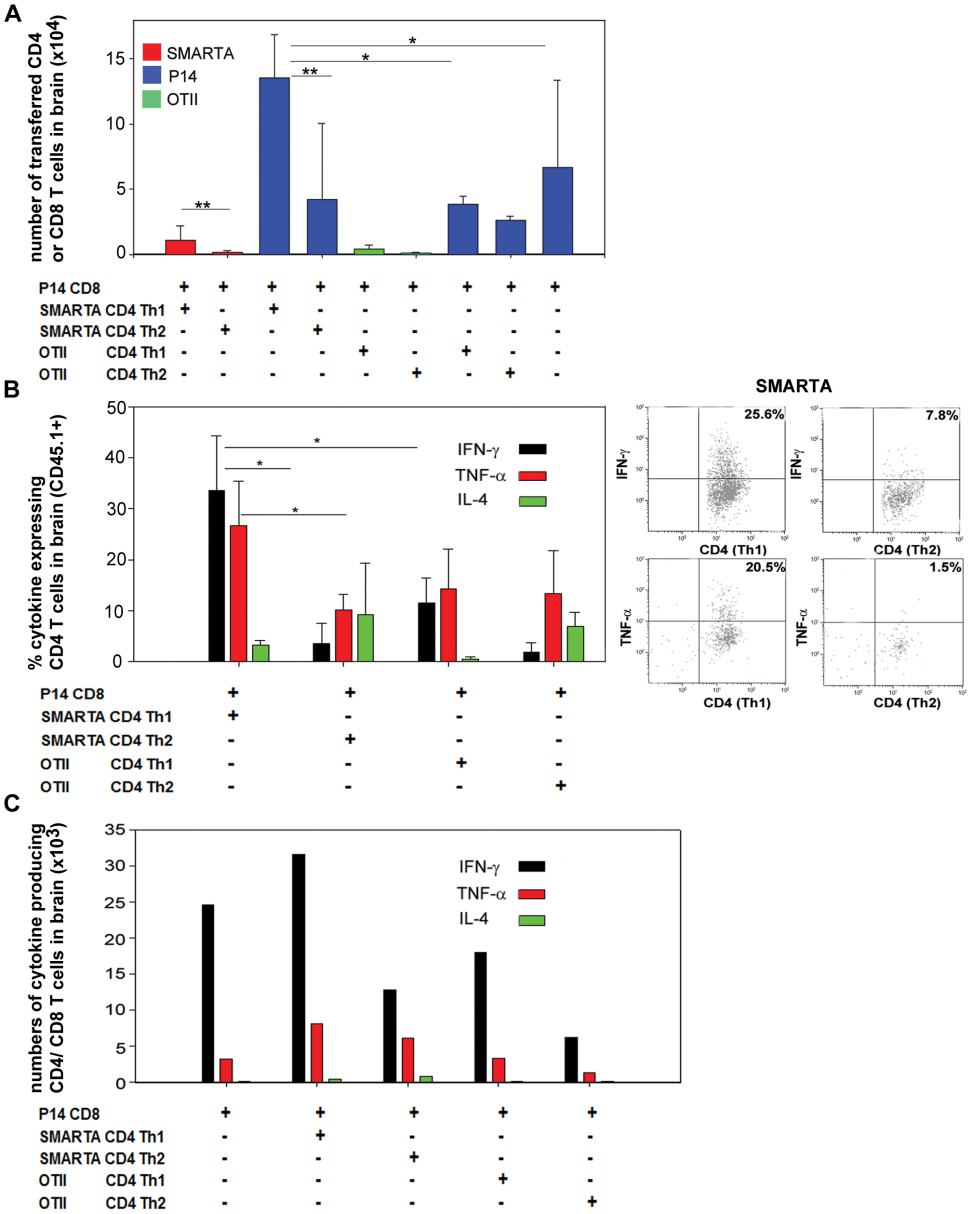
Enhanced recruitment of cytokine expressing T cells to the brain tumour site by co-transfer of tumour-antigen specific CD4 Th1 T cells. Th1 or Th2 polarised T cells from SMARTA and OTII mice were labelled with CFSE. Naïve (non-cultured) P14 CD8 T cells were labelled with Violet dye. The different T cell populations were intravenously transferred into C57BL/6 mice (CD45.2) that had been previously intracranially implanted with 4×10^5^ MC57-GP tumour cells. P14 CD8 T cells were transferred 1 day after tumour implantation; SMARTA and OTII CD4 T cells were transferred the next day, in the combinations indicated. The numbers of cells transferred were 3×10^6^ Th1, 3×10^6^ Th2, and 6×10^6^ P14. At day 6 after tumour implantation, BILs were isolated and analysed ex vivo by multicolour flow cytometry. Adoptively transferred T cells were identified as CD45.1^+^CD4^+^CFSE^+^ cells or CD45.1^+^CD8^+^ Violet dye^+^ cells. Each group of mice comprised 3–5 animals, and results from several experiments were pooled to acquire data from 12 mice. (A) Absolute numbers of each population of adoptively transferred T cells per brain. Results are displayed as means+SD. *P<0.05; **P<0.01: Mann-Whitney Rank sum test. (B) Left panel: intracellular staining of BILs gated on CD45.1^+^CD4^+^CFSE^+^ cells. Expression of IFN-γ, IL-4 and TNF-α is shown after ex vivo restimulation with cognate peptides. Results are displayed as means+SD. *P<0.05: t-test. Right panel: representative dot plots of ex vivo restimulated SMARTA CD4 T cells. (C) Total numbers of cytokine expressing adoptively transferred T cells per brain (including both CD4 and CD8 T cells). Results are displayed as means of 3 pools of mice, from 12 mice in total.

Transfer of CD4 T helper cells was reported to enhance CD8 T cell numbers at the tumour site in other tumour models [28], [31], [32]. We therefore investigated this in the context of Th1 and Th2 CD4 T cells in the intracranial MC57-GP model. This tumour is not only recognised by SMARTA transgenic cells, but also by LCMV-GP specific CD8 T cells from P14 TCR-transgenic mice. Furthermore, we previously demonstrated that adoptively transferred P14 CD8 T cells (without CD4 T cell transfer) can infiltrate intracranial MC57-GP [33]. Here, using mice intracranially implanted with MC57-GP cells, we intravenously transferred either P14 CD8 T cells alone, or P14 followed by transfer of Th1 or Th2 cells (Figure 3); we then sacrificed mice four days post-transfer of CD4 cells for BIL analysis. Consistent with our previous data, CD4 Th1 cells also preferentially accumulated in the brain compared with Th2 cells in this co-transfer setting (Figure 3A, red bars). Moreover, there was a striking P14 CD8 T cell accumulation (blue bars) with co-transfer of Th1 SMARTA cells, which was more than 3 times greater than that achieved with Th2 SMARTA cell transfer. In fact, co-transfer of Th2 SMARTA cells did not significantly alter the baseline accumulation of P14 CD8 cells transferred alone. We also investigated the role of antigen specificity of the CD4 T cells by using co-transfer of OVA-specific OTII Th1 or Th2 CD4 cells, together with tumour antigen-specific P14 CD8 T cells (Figure 3). OTII T cells are antigen non-specific in this experimental model in which the model tumour antigen is LCMV-GP. Although there is brain infiltration of OTII T cells, albeit at a low level (Figure 3A, green bars), the presence of neither Th1 nor Th2 OTII cells augments P14 CD8 T cell infiltration. Thus, both Th1 polarisation and specificity for an antigen present in the recipient mouse are essential for optimal accumulation of CD8 T cells in the brain.

### Tumour-specific Th1 T Cells Modulate the Overall Balance of T-cell Secreted Cytokines at the Tumour Bed

The local anti-tumour immune response in an adoptively transferred immunocompetent host is a combination of direct effects of the transferred cells, and host immune responses. We therefore assessed expression of key cytokines in local immunity at the tumour bed. We had analysed IFN-γ and IL-4 expression on the CD4 Th cells before transfer (Figures 1A-B and S1a); it was then necessary to establish whether transferred T cells would retain the capacity to secrete cytokines several days post adoptive transfer, after infiltrating the brain. We additionally measured TNF-α expression, since it has direct anti-tumour function, and also plays a major role in leukocyte recruitment to the brain [34]. We observed that the proportion of Th1 SMARTA cells able to express IFN-γ in vivo was similar to the levels prior to transfer; they also expressed TNF-α, but little IL-4 (Figure 3B). Th2 SMARTA cells also retained their pre-transfer profile; IL-4 expression was variable, but these data are collected from very low numbers of cells given the feeble infiltration of Th2 cells. Similarly, Th1 and Th2 polarisation profiles are still discernible on the low numbers of in vivo-passaged antigen non-specific OTII CD4 T cells that were analysed. Since the effects for the brain tumour site (and potentially systemically) by adoptive transfer will depend upon both the total number, and function, of infiltrating immune effector cells, we also quantified total numbers of cytokine producing T cells in the brain (Figure 3C). The maximum number of IFN-γ and TNF-α expressing T cells was achieved with co-transfer of SMARTA Th1 cells with P14 CD8 cells.

### Co-transfer of Tumour-antigen Specific CD4 T Cells Enhances Therapeutic Effect of CD8 T cells in a Brain Tumour Model

**Figure 4:**
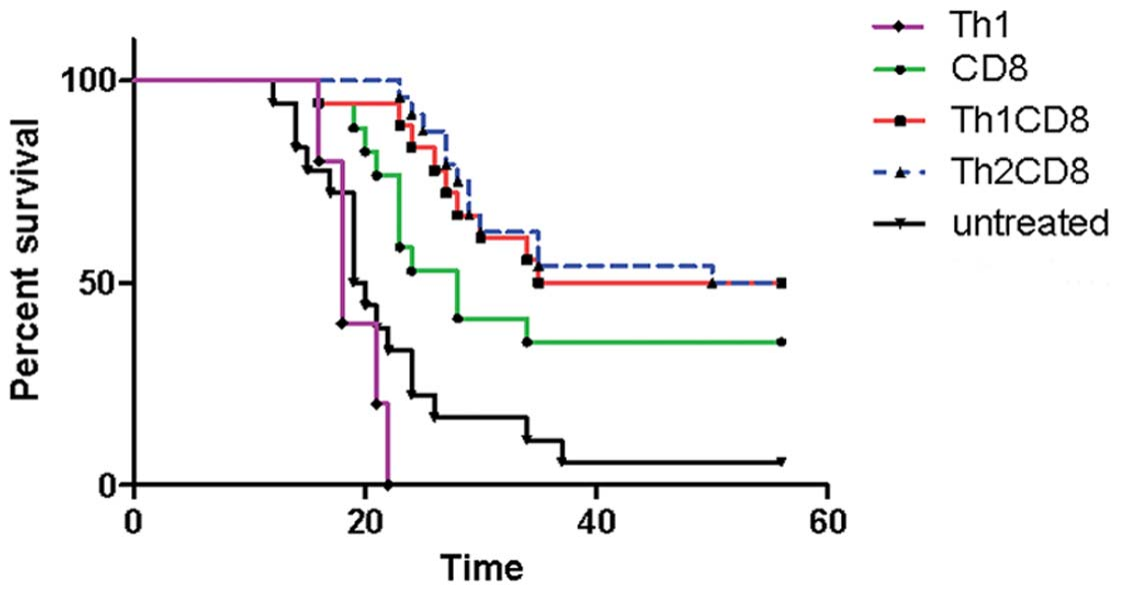
Enhanced survival of brain-tumour bearing mice co-transferred with tumour-antigen specific CD4 and CD8 T cells. In vitro activated OT-I CD8 T cells and polarised OT-II CD4 T cells were intravenously transferred into C57BL/6 mice that had been intracranially implanted with 5×10^5^ EG-7 tumour cells 6 days previously. Groups were CD8 T cells alone: 12×10^6^ OTI T cells; CD8/CD4 Th2∶7×10^6^ OTI T cells +5×10^6^ OTII; CD8/CD4 Th1∶7×10^6^ OTI T cells +5×10^6^ OTII; CD4 Th1 alone: 12×10^6^; untreated: EG-7 tumour cells alone. Mice were monitored until appearance of terminal symptoms (see Methods), at which point they were euthanised. Survival curves represent accumulated data from 2 experiments with 8–12 mice for all groups except CD4 Th1 alone (6 mice). There was a statistically significant difference between CD4 (Th1 or Th2) co-transferred groups and CD8 T cells alone (P<0.05, t-test) at up to 32 days post-implantation of tumour. At the termination of the experiment at 56 days post-implantation, there was a statistically significant difference for mice adoptively transferred with T cells and tumour alone (CD4 Th1 or Th2: P<0.001; CD8 P<0.05, t-test).

Our analyses of T cell cytokine expression and the overall local cytokine milieu suggested that co-transfer of Th1 CD4 T cells with CD8 T cells would have a significant anti-tumour effect. We therefore tested this in the EG-7 tumour model, in which there is expression of the OVA antigen recognised by CD4 and CD8 T cells available from OTII and OTI TCR transgenic mice, respectively. This model was chosen for testing because unlike for MC57-GP tumours, adoptive transfer of CD8 T cells alone has only modest therapeutic efficacy, and transfer of Th1 polarised CD4 T cells alone had no therapeutic effect (Figure 4). EG-7 tumours were established in syngeneic mice by intracranial implantation, then after 6 days, mice were either left untreated or were intravenously infused with in vitro activated CD8 OTI T cells alone or with activated Th1 or Th2 OTII T cells (Figure 4). To stringently test whether any therapeutic effect was correlated with the total number of T cells transferred, or their functional phenotype, we kept total cell number constant (detailed in legend). At 25 days post tumour implantation, more than 50% of untreated mice had terminal symptoms and were sacrificed, and by the end of the experiment (day 56) only 5.6% of untreated mice survived. Untreated mice had a median survival of 19.5 days; this was extended to 28 days by adoptively transferred antigen-specific CD8 T cells alone. The additional transfer of CD4 Th1 or Th2 T cells increased the survival significantly, with more than 50 percent of mice surviving 56 days post implantation, with a median survival of 46 and 53 days, respectively. Surprisingly we did not observe a significant difference in survival between mice receiving Th1 or Th2 cells (co-transferred with the CD8 T cells); both polarisations were effective. In view of this finding, we tested whether tumour implanted mice adoptively transferred with Th2 cells alone had significantly prolonged survival compared with untreated mice: this was not the case (Figure S4). Overall, we demonstrate that the inclusion of tumour antigen specific CD4 T cells for adoptive immunotherapy of brain tumours is more efficacious than transferring an equivalent number of CD8 T cells alone.

## Discussion

The enormous strides in tumour immunology and its application in cancer treatment have entered a new era based on proven efficacy in Phase III clinical trials for certain malignancies. Indeed, as aptly stated in a recent state-of-the-art review, cancer immunotherapy can be considered to have come of age [1]. Nevertheless, immunotherapy for tumours of the CNS presents special challenges, but these are gradually being met. The potential of brain tumour immunotherapy was highlighted in pioneering clinical studies more than a decade ago [35]. With advances in brain tumour immunobiology, there are now opportunities to update the approach by targeting newly defined glioma antigenic targets [36], [37], [38]. Moreover, for primary CNS lymphoma, those arising in immunosuppressed patients are generally positive for Epstein Barr Virus, which can offer a suitable target for therapeutic T cells [39]. However, further rational development and optimisation of CNS cancer immunotherapy requires knowledge of immune cell function at the tumour site in the brain, information that is difficult to obtain from clinical studies. The data we present in this study from animal models is therefore of particular translational relevance.

As predicted from previous studies on CD4 T cell biology [26], [40], differential polarisation status induced different patterns of chemokine receptors and adhesion molecules. In our study, preferential homing of Th1 CD4 T cells to intracranially implanted tumours correlated with high expression of CXCR3, which is a receptor for IFN-γ-inducible protein (IP)-10/CXCL10. This result is consistent with previous observations for type 1 (Tc1) polarised CD8 T cells, which efficiently infiltrated brain tumours in a CXCL10 dependent manner [16], [41]. We also observed significant expression of CD49d (α4 integrin) on Th1 CD4 cells. Our polarised cells also expressed β1 integrin (CD29), but little or no α4β7 integrin (data not shown). Thus, as α4 can only pair with β1 and β7 integrins, these cells most likely expressed VLA-4 (α4β1), which is an adhesion molecule well characterised in both murine [15], [16], [17] and human studies [18]. VLA-4 facilitates CNS trafficking of T cells by binding to vascular cell adhesion molecule 1 (VCAM-1) or fibronectin on the blood brain barrier. Indeed, targeting α4-integrin with a blocking antibody in patients with multiple sclerosis suppressed CNS accumulation of CD4 T cells and reduced the risk of the rate of clinical relapse [18]. It should be noted that in our in vitro polarisation experiments, there was still CD49d expression on the Th2 cells that hardly entered the brain, even though the levels were slightly lower than on Th1 cells. It is therefore likely that VLA-4 expression is necessary, but not sufficient to ensure efficient entry to the tumour implanted brain. Indeed, homing receptors responsible for directing T cells to all tissues are not fully elucidated, and in the case of malignancy, tumour associated vasculature may differ from that of healthy tissues [42].

Our study illustrated major functional consequences of CD4 T cell infiltration into intracranial tumours that augmented CD8 T cell recruitment, the local cytokine milieu, and ultimately anti-tumour immunity. These effects were observed principally when the CD4 T cells were specific for a tumour expressed antigen, indicating that recognition of MHC class II restricted cognate antigen was necessary at the tumour site in the brain. The EG-7 and MC57-GP tumour models that we used in our study are both MHC class II^−^ (data not shown). However, we cannot exclude that there is MHC class II induction on tumour cells in vivo, particularly in the context of strong local IFN-γ expression that we measured. But tumour antigens can also be presented by antigen presenting cells (APCs) in the tumour stroma, as we have previously demonstrated for cross-presenting APC and retention of CD8 T cells in another brain tumour model [43]. Moreover, tumour antigen-MHC class I complexes were directly identified on CD11b^+^ brain tumour stromal cells [44]. Since antigen presentation to CD4 T cells is less stringent than cross-presentation to CD8 T cells, this function is likely to be readily achieved by APC present in the tumour bearing mouse brain [45]. However, in the context of human glioblastoma, immune function of local APC may be compromised, and the full potential of CD4 T cells functioning in the tumour bed may thus require concomitant modulation of the tumour microenvironment [46], [47]. Once CD4 T cells are reactivated at the tumour site, their expression of IFN-γ and TNF-α that we measured has multiple potential anti-tumoural consequences. However, since Th1 CD4 cell transfer alone did not have therapeutic effect in the EG-7 tumour model, the local concentration of these Th1 cytokines may have been insufficient for direct anti-tumour function, but could still amplify the inflammatory response. Indeed, CD4 enhancement of CD8 T cell infiltration was previously described for extracranial tumours in elegant studies by Sherman and colleagues, in which they showed that tumour antigen-specific CD4 T cells rendered the tumour microenvironment permissive for CD8 T cell entry and function through IFN-γ-dependent chemokine induction [31], [32]. Such a function would be consistent with our findings for intracranial tumours. We also noted elevated TNF-α expression in the infiltrate of mice transferred with Th1 cells. TNF-α has many effects on brain vasculature that can directly promote immune cell infiltration, including an increase in blood brain barrier permeability [48], and an increase in VCAM-1 expression [49].

The equally beneficial roles of Th1 and Th2 CD4 cells in our long term tumour therapy experiments were unexpected in view of the very clear advantages for Th1 transfer in short term experiments. It is possible that over the 56 days of the experiment there is eventually sufficient accumulation of Th2 cells at the tumour site to have a therapeutic effect, although we were unable to isolate or reproducibly identify adoptively transferred cells a long time after the initial transfer (data not shown). However, it has previously been reported that Th2 cells can have anti-tumour activity [20], [21], possibly through IL-4 and the recruitment of innate immune cells [50]. It is also possible that the Th2 cells that are initially transferred are restimulated and repolarised in vivo, to express Th1 cytokines which ultimately exert therapeutic effect. In support of this hypothesis, we demonstrated that CD4 OTII Th2 polarised cells could be repolarised in vitro (under Th1 polarising conditions) to express significant IFN-γ, as well as CXCR3 on a proportion of the cells (Figure S5). The IFN-γ expressing T cells also mostly co-expressed IL-4, suggesting that they were repolarised IL-4 secreting Th2 cells, rather than an outgrowth of Th1 cells that were in the original culture. It should be noted that in vivo, with co-transfer of CD8 T cells (plus any endogenous type 1 immune cells), there is likely to be significant IFN-γ available to potentially reproduce the cytokine balance we created in vitro. Indeed, in vivo reprogramming of Th2 cells to become “Th2+1″ cells, expressing both IL-4 and IFN-γ has been previously reported in the context of viral infection [51], although not, to our knowledge, in the case of malignancy.

Overall, our study firmly establishes the benefit of incorporating tumour specific CD4 T cells in adoptive cell therapies for brain tumours. Although not demonstrated in our study, it is probable that in other forms of immunotherapy such as cancer vaccines, there could be additional benefits of CD4 cells based on their capacity to act on dendritic cells and promote CD8 expansion, function, and memory induction [52]. Existing cancer vaccination approaches have employed either tumour expressed CD4 epitopes, or “universal” CD4 epitopes, in an attempt to provide overall T cell help [53]. The latter approach, using antigens such as keyhole limpet haemocyanin, Pan DR helper T cell epitope (PADRE), or tetanus toxoid, is readily applicable to any patient or malignancy. However, our data, at least for brain tumours, suggests that optimal benefit from CD4 T cells will be achieved by choosing a tumour-expressed antigen, which will ensure antigen-specific restimulation of the CD4 T cells at the tumour site. Identification of further tumour antigens recognised by CD4 T cells will therefore maximise opportunities of designing efficacious cancer immunotherapies.

## Supporting Information

Figure S1 **Distinct phenotypes of OTII CD4 T cells activated under Th1 and Th2 polarising conditions.** (a) Representative histogram of intracellular staining of polarised T cells for IFN-γ and IL-4 after PMA and ionomycin activation. (b) Representative histogram of surface staining for cell surface receptors implicated in lymphocyte homing; chemokine receptors CXCR3, CCR4, and CD49d (or isotype control).
(TIF)

Figure S2 **Gating strategy for identification of adoptively transferred CD4 T cells.** T cells from OTII (CD45.1) mice were polarised, then Th1 were labelled with CFSE and Th2 were labelled with Violet dye. T cells were injected in a 1∶1 ratio into the same recipient. After 19 or 96 hours (according to the experiment), BILs were isolated (see Methods) and surface stained for the CD45.1 congenic marker, gated on CD4^+^ CD45.1^+^ live cells. Gate V was then used for identification of adoptively transferred cells, and CFSE or Violet Dye was used to distinguish the differentially in vitro polarised Th1 and Th2 cells and to gate them for further analysis.
(TIF)

Figure S3 **Adoptively transferred OTII Th1 cells show preferential homing compared to Th2 cells.** Cell suspensions were prepared from lymph nodes and spleen of OTII mice and activated under Th1 or Th2 polarising conditions (see Methods). OTII T cells (CD45.1) were labelled with CFSE (Th1) or Violet dye (Th2) and were intravenously transferred (3×10^6^ Th1; 3×10^6^ Th2) into C57BL/6 mice (CD45.2) that had been intracranially implanted with 5×10^5^ EG-7 cells 6 days previously. After 19 hours BILs were isolated, stained with antibodies for CD4 and for CD45.1 and were analysed ex vivo by multicolour flow cytometry. Adoptively transferred T cells were identified as CD45.1^+^CD4^+^ cells that were either CFSE^+^ or Violet dye^+^. Results are expressed as the percentage of Th1 and Th2 cells among the adoptively transferred CD45.1^+^CD4^+^ cells in the BILs, each symbol represents an individual mouse.
(TIF)

Figure S4 **No survival advantage of brain-tumour bearing mice treated by adoptive transfer of tumour-antigen specific CD4 Th2 cells alone.** In vitro activated and Th2 polarised OTII CD4 T cells were intravenously transferred into C57BL/6 mice that had been intracranially implanted with 5×10^5^ EG-7 tumour cells 6 days previously. Groups were either untreated mice or 12×10^6^ CD4 Th2 alone. Mice were monitored until appearance of terminal symptoms (see Methods), at which point they were euthanised. Survival curves represent data from 6 mice/group.
(TIF)

Figure S5 **OTII CD4 T cells activated under Th2 polarising conditions can be repolarised in vitro.** Cell suspensions were prepared from lymph nodes and spleen of OTII mice and activated under Th2 polarising conditions for 10 days. Culture medium was then replaced with medium promoting Th1 polarisation (see Methods). At day 14, OTII cells were restimulated with irradiated spleen cells and peptide under Th1 polarising conditions. Medium was replaced according to cell proliferation (every 2–3 days). Intracellular staining was performed with isotype control antibodies (left dot plots) or cytokine specific antibodies (right dot plots) at the indicated days (A), and surface staining was performed using isotype control antibodies (black curves) or CCR4 and CXCR3 specific antibodies (red curves) (B). Figures on the dot plots represent percentage of cells in each quadrant, positioned according to isotype staining. All stainings shown are on live-gated CD4^+^ cells.
(TIF)
