# Supplementary figures and images for: Synergy between CD8 T Cells and Th1 or Th2 Polarised CD4 T Cells for Adoptive Immunotherapy of Brain Tumours
Source: PLoS One. 2013 May 23;8(5):e63933. doi: 10.1371/journal.pone.0063933 (PMC3662716; doi:10.1371/journal.pone.0063933)

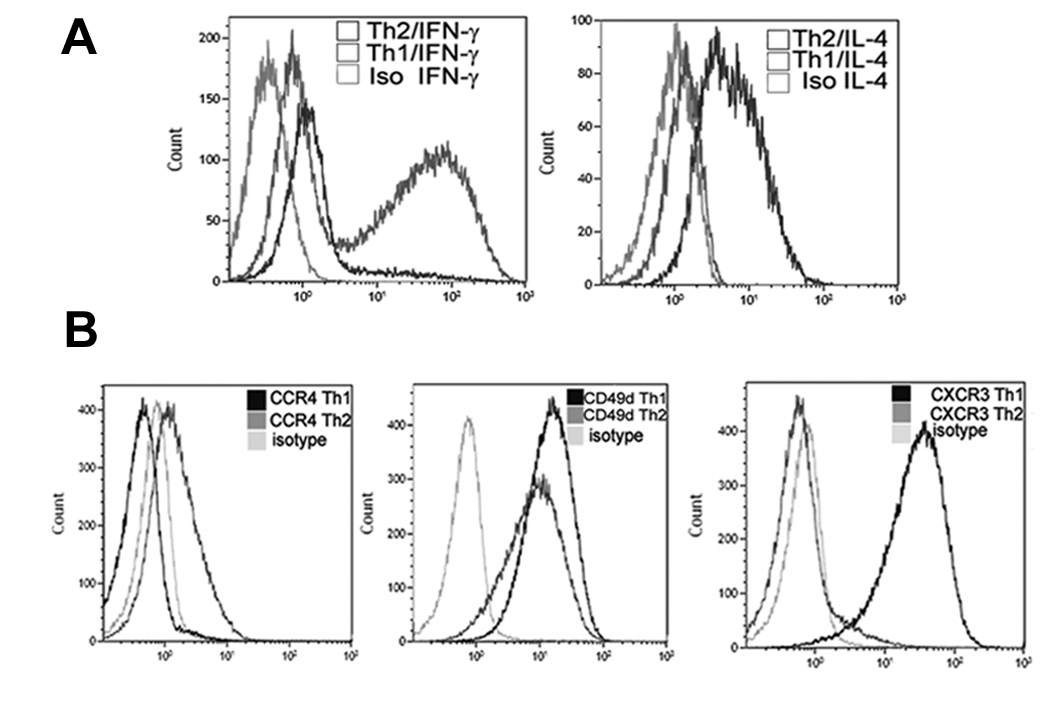

Supplement: Figure S1 — Distinct phenotypes of OTII CD4 T cells activated under Th1 and Th2 polarising conditions. (a) Representative histogram of intracellular staining of polarised T cells for IFN-γ and IL-4 after PMA and ionomycin activation. (b) Representative histogram of surface staining for cell surface receptors implicated in lymphocyte homing; chemokine receptors CXCR3, CCR4, and CD49d (or isotype control). (TIF) [file pone.0063933.s001.tif]

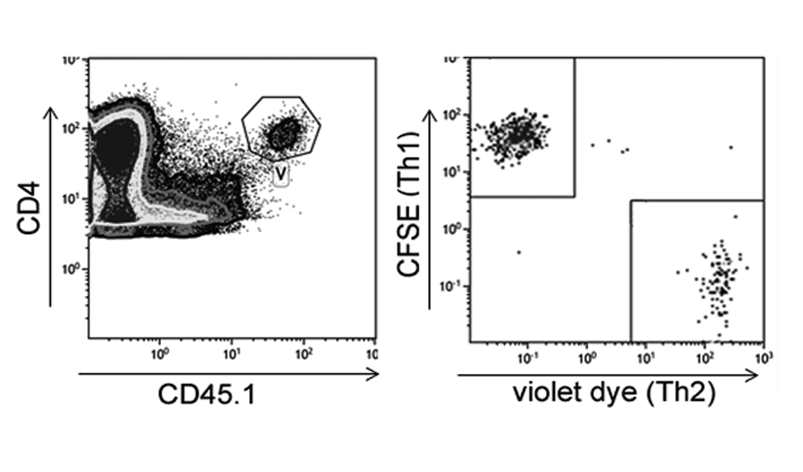

Supplement: Figure S2 — Gating strategy for identification of adoptively transferred CD4 T cells. T cells from OTII (CD45.1) mice were polarised, then Th1 were labelled with CFSE and Th2 were labelled with Violet dye. T cells were injected in a 1∶1 ratio into the same recipient. After 19 or 96 hours (according to the experiment), BILs were isolated (see Methods) and surface stained for the CD45.1 congenic marker, gated on CD4+ CD45.1+ live cells. Gate V was then used for identification of adoptively transferred cells, and CFSE or Violet Dye was used to distinguish the differentially in vitro polarised Th1 and Th2 cells and to gate them for further analysis. (TIF) [file pone.0063933.s002.tif]

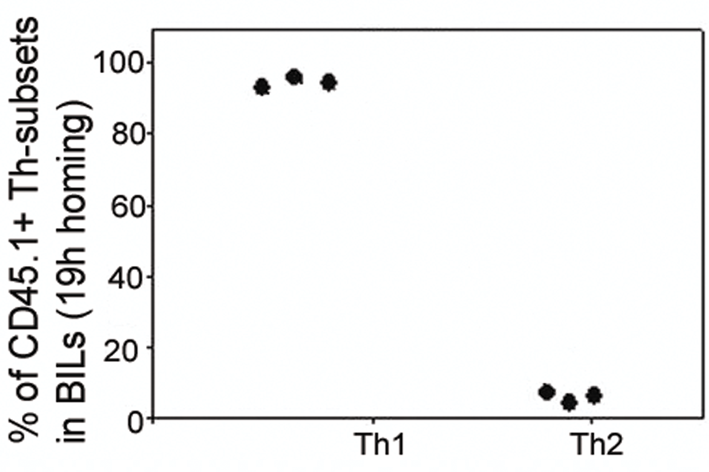

Supplement: Figure S3 — Adoptively transferred OTII Th1 cells show preferential homing compared to Th2 cells. Cell suspensions were prepared from lymph nodes and spleen of OTII mice and activated under Th1 or Th2 polarising conditions (see Methods). OTII T cells (CD45.1) were labelled with CFSE (Th1) or Violet dye (Th2) and were intravenously transferred (3×10^6^ Th1; 3×10^6^ Th2) into C57BL/6 mice (CD45.2) that had been intracranially implanted with 5×10^5^ EG-7 cells 6 days previously. After 19 hours BILs were isolated, stained with antibodies for CD4 and for CD45.1 and were analysed ex vivo by multicolour flow cytometry. Adoptively transferred T cells were identified as CD45.1+CD4+ cells that were either CFSE+ or Violet dye+. Results are expressed as the percentage of Th1 and Th2 cells among the adoptively transferred CD45.1+CD4+ cells in the BILs, each symbol represents an individual mouse. (TIF) [file pone.0063933.s003.tif]

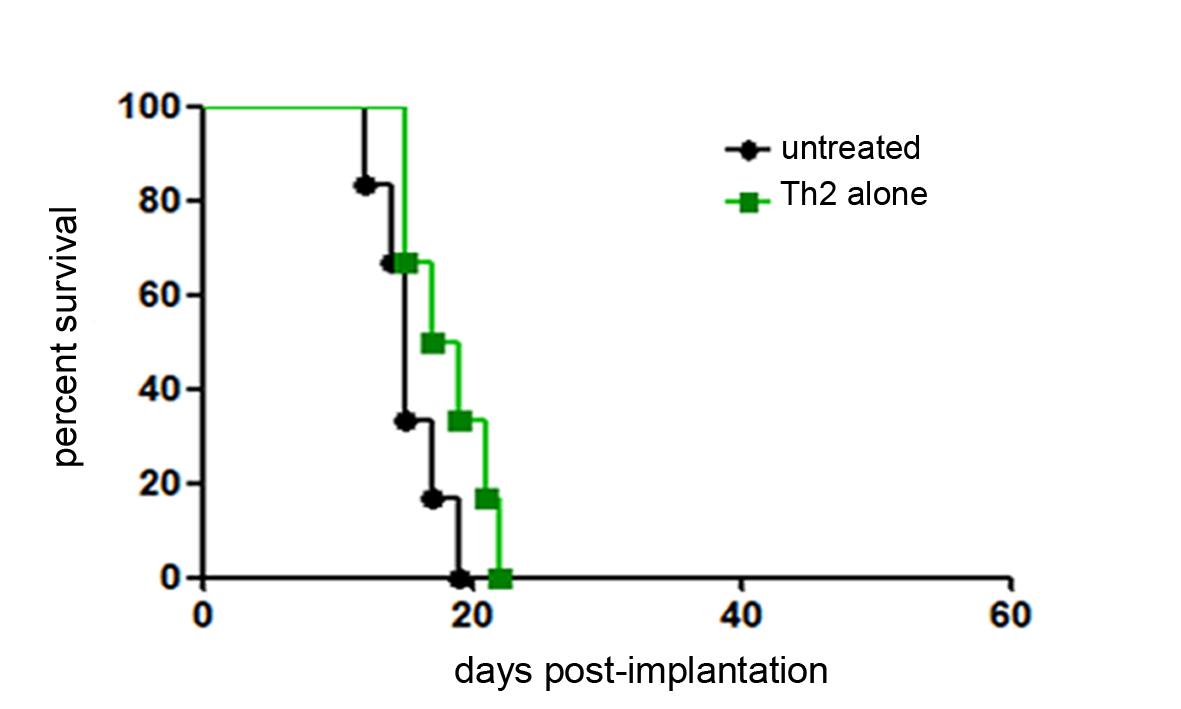

Supplement: Figure S4 — No survival advantage of brain-tumour bearing mice treated by adoptive transfer of tumour-antigen specific CD4 Th2 cells alone. In vitro activated and Th2 polarised OTII CD4 T cells were intravenously transferred into C57BL/6 mice that had been intracranially implanted with 5×10^5^ EG-7 tumour cells 6 days previously. Groups were either untreated mice or 12×106 CD4 Th2 alone. Mice were monitored until appearance of terminal symptoms (see Methods), at which point they were euthanised. Survival curves represent data from 6 mice/group. (TIF) [file pone.0063933.s004.tif]

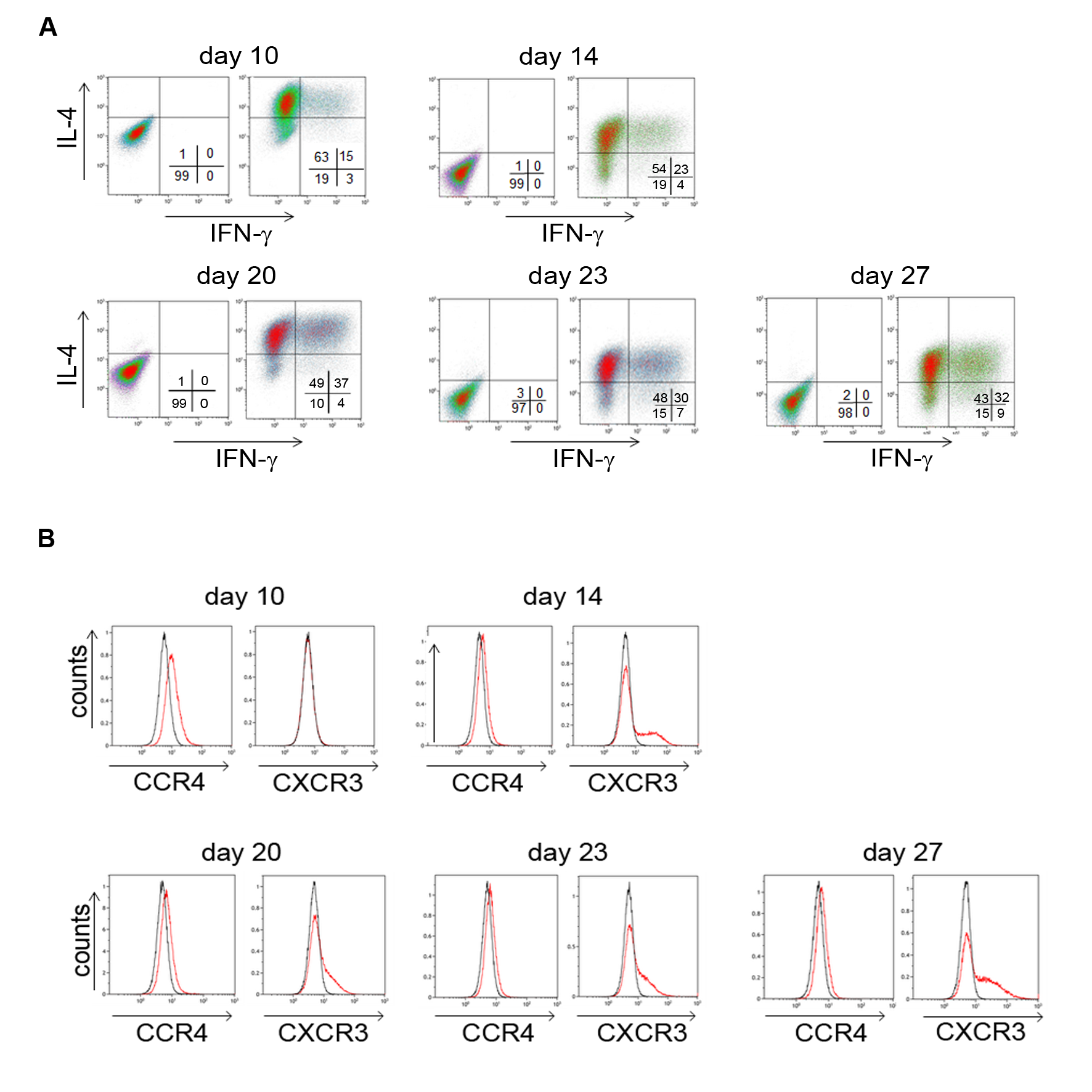

Supplement: Figure S5 — OTII CD4 T cells activated under Th2 polarising conditions can be repolarised in vitro. Cell suspensions were prepared from lymph nodes and spleen of OTII mice and activated under Th2 polarising conditions for 10 days. Culture medium was then replaced with medium promoting Th1 polarisation (see Methods). At day 14, OTII cells were restimulated with irradiated spleen cells and peptide under Th1 polarising conditions. Medium was replaced according to cell proliferation (every 2–3 days). Intracellular staining was performed with isotype control antibodies (left dot plots) or cytokine specific antibodies (right dot plots) at the indicated days (A), and surface staining was performed using isotype control antibodies (black curves) or CCR4 and CXCR3 specific antibodies (red curves) (B). Figures on the dot plots represent percentage of cells in each quadrant, positioned according to isotype staining. All stainings shown are on live-gated CD4+ cells. (TIF) [file pone.0063933.s005.tif]
